# Glucose‐lowering medication associated with weight loss may limit the progression of diabetic neuropathy in type 2 diabetes

**DOI:** 10.1111/jns.12664

**Published:** 2024-10-22

**Authors:** Georgios Ponirakis, Ibrahim Al‐Janahi, Einas Elgassim, Rawan Hussein, Ioannis N. Petropoulos, Hoda Gad, Adnan Khan, Hadeel B. Zaghloul, Mashhood A. Siddique, Hamda Ali, Fatima F. S. Mohamed, Lina H. M. Ahmed, Youssra Dakroury, Abeer M. M. El Shewehy, Ruba Saeid, Fadwa Mahjoub, Shaikha N. Al‐Thani, Farheen Ahmed, Moayad Homssi, Salah Mahmoud, Nebras H. Hadid, Aisha Al Obaidan, Iuliia Salivon, Ziyad R. Mahfoud, Mahmoud A. Zirie, Yousuf Al‐Ansari, Stephen L. Atkin, Rayaz A. Malik

**Affiliations:** ^1^ Department of Medicine Weill Cornell Medicine‐Qatar, Qatar Foundation Doha Qatar; ^2^ National Diabetes Center, Hamad General Hospital Hamad Medical Corporation Doha Qatar; ^3^ Royal College of Surgeons in Ireland Bahrain Adliya Bahrain; ^4^ Division of Cardiovascular Sciences, Faculty of Biology, Medicine and Health University of Manchester Manchester UK; ^5^ Faculty of Science and Engineering Manchester Metropolitan University Manchester UK

**Keywords:** corneal confocal microscopy, diabetic peripheral neuropathy, glucose lowering medication, type 2 diabetes, weight change

## Abstract

**Aim:**

Obesity is a major risk factor for diabetic peripheral neuropathy (DPN) in type 2 diabetes (T2D). This study investigated the effect of glucose lowering medication associated with weight change on DPN.

**Methods:**

Participants with T2D were grouped based on whether their glucose lowering medications were associated with weight gain (WG) or weight loss (WL). They underwent clinical, metabolic testing and assessment of neuropathic symptoms, vibration perception threshold (VPT), sudomotor function and corneal confocal microscopy (CCM) at baseline and follow‐up between 4 and 7 years.

**Results:**

Of 76 participants, 69.7% were on glucose lowering medication associated with WG, and 30.3% were on glucose lowering medication associated with WL. At baseline, participants in the WG group had a significantly longer duration of diabetes (*p* < .01), higher douleur neuropathique en 4 (DN4) score (*p* < .0001) and VPT (*p* = .01) compared with those in the WL group. Over a 56‐month period, participants in the WG group showed no significant change in body weight (*p* = .11), HbA1c (*p* = .18), triglycerides (*p* = .42), DN4 (*p* = .11), VPT (*p* = .15) or Sudoscan (*p* = .43), but showed a decline in corneal nerve fiber density (CNFD), corneal nerve branch density (CNBD) and corneal nerve fiber length (CNFL) (*p* < .0001). Participants in the WL group showed a reduction in weight (*p* = .01) and triglycerides (*p* < .05), no change in DN4 (*p* = .30), VPT (*p* = .31) or Sudoscan (*p* = .17) and a decline in the corneal nerve branch density (*p* < .01).

**Conclusions:**

Participants treated with glucose lowering medication associated with weight gain had worse neuropathy and greater loss of corneal nerves during follow‐up, compared to patients treated with medication associated with weight loss.

## 1 INTRODUCTION

Around half of all people with diabetes are affected by diabetic peripheral neuropathy (DPN), which can cause neuropathic pain, erectile dysfunction, and foot ulceration.^1^ While intensive glycemic control can delay or prevent DPN in type 1 diabetes (T1D),^2^ there is conflicting data on its effectiveness in type 2 diabetes (T2D).^3^, ^4^, ^5^, ^6^, ^7^, ^8^, ^9^ Obesity is a primary risk factor for DPN in T2D.^10^ Studies have shown that modest weight loss (WL) can prevent or improve DPN in people with T2D,^11^, ^12^ but until recently, most glucose lowering medications were associated with weight gain (WG). The American Diabetes Association (ADA) has recommended using glucose lowering medications that do not promote WG and promote WL for overweight or obese people with T2D.^13^

A large proportion of glucose lowering medications used for T2D, for example, insulin, sulfonylurea (SU), and thiazolidinedione (TZD), are associated with WG.^14^ WG occurs due to increased lipogenesis in muscle and adipose tissues in those on insulin, with defensive snacking to avoid hypoglycemia in those on SU and through enhancing the storage of fat in adipocytes, increasing appetite, and fluid retention in those on TZD.^14^ Glucose lowering medications associated with WL include metformin, glucagon‐like peptide‐1 (GLP‐1) receptor agonists, and sodium glucose co‐transporter 2 (SGLT2) inhibitors.^14^ WL is likely related to anorectic effects in those on metformin, delayed gastric emptying and reduced appetite in those on GLP‐1 receptor agonists, and a calorie deficit and fluid loss in those on SGLT2 inhibitors.^14^ The Bypass Angioplasty Revascularization Investigation 2 Diabetes (BARI‐2D) sub‐study of 1075 participants without DPN showed that the 4‐year cumulative incidence of DPN was significantly lower in the insulin‐sensitizing (metformin and TZD) compared with the insulin‐providing (insulin and SU) strategy, even after adjustment for in‐trial HbA1c.^5^

The predictive utility of corneal confocal microscopy (CCM) for incident DPN has been established in two independent studies.^15^, ^16^ We previously showed that people with T2D treated with glucose lowering medication associated with WG showed a decrease in corneal nerve branch density over 2 years.^11^ In an intervention study, we also showed that a combination of pioglitazone with exenatide once weekly or basal bolus insulin was associated with corneal nerve regeneration, but no change in vibration perception threshold or sudomotor function, despite weight gain in both groups.^17^ This study investigated whether the use of glucose lowering medications associated with WG or WL was associated with a worsening or improvement in neuropathic symptoms, vibration perception, sudomotor function, and CCM.

## 2 METHODS

### 2.1 Project design

Participants with T2D were recruited from the National Diabetes Center in Hamad General Hospital in Qatar between January 2017 and October 2022. The study obtained ethics approval from the WCM‐Q IRB (#14‐00058 and 20‐00024) and HMC IRB (#15103/15 and MRC‐01‐21‐386) and adhered to the principles of the Declaration of Helsinki. All participants provided informed consent prior to enrolment. Participants or the public were not involved in the design, or conduct, or reporting, or dissemination plans of our research.

### 2.2 Participant selection

Eligible participants were aged between 18 and 80 years and were excluded if they had a history of allergy to Oxybuprocaine, a local anesthetic used for the CCM, conditions that may affect corneal nerve morphology such as severe chronic dry eyes, a history of injury or surgery to the eye in the preceding 12 months. Exclusion criteria also included renal failure (CKD stages 4 and 5), medications leading to insulin resistance (e.g., corticosteroids), pregnancy, active retinopathy, and any causes of neuropathy other than diabetes, including Sjogren's syndrome, systemic lupus erythematosus, HIV, hepatitis B and C, inherited neuropathies, tumors, and alcoholism. Participants underwent assessments at baseline and follow‐up between 4 and 7 years.

### 2.3 Independent variables

The study categorized medications based on their known association with weight change at baseline. One group included medications associated with weight gain (WG), for example, insulin, thiazolidinediones, and sulfonylureas, and the other group included medications associated with weight loss (WL), for example, metformin, glucagon‐like peptide‐1 (GLP‐1) receptor agonists, and sodium‐glucose co‐transporter 2 (SGLT2) inhibitors. Participants who were on both WG and WL medications were categorized under the WG medication group to isolate the impact of medications associated with WL. Participants in the WL group only took WL medications throughout the follow‐up.

### 2.4 Dependent variables

Corneal confocal microscopy (CCM) was performed using the Heidelberg Retina Tomograph and the Rostock Cornea Module (Heidelberg Engineering GmbH, Heidelberg, Germany).^18^ The cornea was anesthetized using a drop of oxybuprocaine hydrochloride 0.4% (Chauvin Pharmaceuticals, Chefaro, UK). Viscotears gel (Carbomer 980, 0.2%, Novartis, UK) was applied on the front of the eye as the coupling agent between the cornea and the cap on the CCM. The participant was instructed to fixate on a target with the eye not being examined. Several scans of the sub‐basal nerve plexus in the central cornea were captured per eye for approximately 2 min. The field of view of each image is 400 × 400 μm. At a separate time, three high clarity images per eye of the sub‐basal nerve plexus were selected by one researcher blind to the participant's health condition. Criteria for image selection are depth, focus position, and contrast.^19^ Three corneal nerve measures: corneal nerve fiber density (CNFD) (number of main nerve fibers/mm^2^), corneal nerve branch density (CNBD) (number of branches/mm^2^), and corneal nerve fiber length (CNFL) (length of main nerves and branches mm/mm^2^) were quantified manually using CCMetrics.^20^

Vibration perception threshold (VPT) was measured on the pulp of the large toe with a Neurothesiometer (Horwell, Scientific Laboratory Supplies, Wilford, Nottingham, UK).^21^ The test was repeated three times and the average value was recorded.

Sudomotor function was measured using Sudoscan (Impeto Medical SAS) as described previously.^22^ Sudoscan evaluates sympathetic innervation based on sweat chloride concentrations generated by the sweat gland in response to the voltage applied and is reported as electrochemical skin conductance (ESC) in microSiemens (μS).

The douleur neuropathique en 4 (DN4) questionnaire was used to identify neuropathic symptoms, including burning pain, painful cold sensations, electric shock‐like sensations, tingling, pins and needles, numbness, and itching.^23^, ^24^

### 2.5 Covariates

Clinical and metabolic measures, including age, diabetes duration, body mass index (BMI), systolic (SBP) and diastolic blood pressure (DBP), HbA1c, and lipid profile, were recorded from the electronic medical register (Cerner).

### 2.6 Power calculation

This is a 56‐month extension study of a 24‐month longitudinal study on the same cohort of 76 subjects with T2D.^11^ In the original study, there was a significant change in CNFL from 17.8 ± 6.2 mm/mm^2^ at baseline to 16.4 mm/mm^2^ at follow‐up. In a retrospective power analysis based on the observed effect size, standard deviation, and sample size from the original study, utilizing a two‐tailed paired *t*‐test, with an *α* level set at .05, the available sample size of 76 subjects yielded an estimated power of approximately 71.57%. This indicates a moderate to high likelihood of detecting a significant change in CNFL assuming that the effect size and standard deviation remained consistent with those observed in the original 24‐month study. The longer duration of follow‐up in this extension study could potentially enhance the power to detect clinically meaningful changes in CNFL over time.

### 2.7 Data analysis and statistics

Variables were summarized using mean and standard deviation. The independent *t*‐test was used to compare the variables at baseline between those in the WG and WL groups to compare the differences in the change in parameters from baseline to the last visit between the two groups. A paired *t*‐test was used for within‐group comparisons of variables between baseline and last visit, restricted to those with complete datasets at both time points, representing a smaller subset compared with the independent *t*‐test, which includes all baseline measurements. To determine if differences in change of the neuropathy measures between the WG and WL groups were statistically significant, an analysis of covariance (ANCOVA) was conducted to adjust for baseline neuropathy measures, variation in follow‐up time, and the duration of diabetes, which were significantly different between the treatment groups.

Bivariate linear regression analysis was performed to assess the associations between change (Δ) in CNFD, ΔCNBD, and ΔCNFL as the dependent variables, with medications as the independent variable. Age, diabetes duration, Δblood pressure, ΔBMI, ΔHbA1c, Δcholesterol, Δtriglyceride, ΔHDL, and ΔLDL were included as confounders. All dependent variables were normally distributed as assessed by Q‐Q plots and histograms. Dependent variables that were significant on the bivariate level were included in the multiple linear regression analysis. Results are reported as regression coefficients with 95% confidence intervals.

All analyses were performed using IBM‐SPSS (version 26; SPSS Inc., Armonk NY) with a *p* value of <.05 considered as statistically significant.

## 3 RESULTS

Of 76 participants with T2D enrolled in the study, 53 (69.7%) were taking glucose lowering medication associated with weight gain (WG), while 23 (30.3%) were taking glucose lowering medication associated with weight loss (WL) (Table 1). Follow‐up assessments were performed between 42 and 78 months from baseline.

**TABLE 1:** Frequency of glucose lowering medications associated with weight change.

	Participants (*n*/76)	Percent
Glucose lowering medications associated with weight gain		
Insulin	44	57.9
Thiazolidinediones (pioglitazone)	6	7.9
Sulfonylureas (glimepiride)	2	2.6
Glucose lowering medications associated with weight loss		
Metformin	61	80.3
GLP‐1 agonists (dulaglutide, exenatide, semaglutide, liraglutide)	19	25.0
SGLT2 inhibitors (dapagliflozin or empagliflozin)	47	61.8
Medications associated with weight gain	53	69.7
Medications associated with weight loss	23	30.3

*Note*: Patients on weight loss (WL) associated glucose lowering (GL) medications in combination with weight gain (WG) associated GL medications were categorized in the WG medication group. Percentages exceed 100% because some patients may be on more than one medication.

### 3.1 Comparison of clinical characteristics between WG and WL groups

At baseline (Table 2), the WG and WL groups had comparable age (55.3 ± 9.1 vs. 54.9 ± 7.5 years, *p* = .43), sex (52.2% vs. 34.6% male, *p* = .15), HbA1c (*p* = .21), total cholesterol, triglyceride, HDL, LDL (*p* = .32, .43), systolic and diastolic blood pressure (*p* = .16), body weight and BMI (*p* = .18–.26), and eGFR and creatinine (*p* = .56, .60). However, those in the WG group had a significantly longer diabetes duration compared with those in the WL group (15.4 ± 7.0 vs. 10.5 ± 7.1 years, *p* < .01).

**TABLE 2:** Comparison of baseline clinical and neuropathy characteristics between participants with type 2 diabetes on medications associated with weight loss and gain.

	Weight loss group (*n* = 23)	Weight gain group (*n* = 53)	*p* value
Age, years	55.3 ± 9.1	54.9 ± 7.5	.43
Men and women, %	52.2 and 47.8	34.6 and 65.4	.15
Diabetes duration, years	10.5 ± 7.1	15.4 ± 7.0	<.01
HbA1c, mmol/mol	63.9 ± 11.5	66.2 ± 9.3	.21
Hb1Ac, %	8.0 ± 1.0	8.2 ± 0.9	.21
Total cholesterol baseline, mmol/L	4.3 ± 1.2	4.2 ± 1.1	.43
Triglyceride baseline, mmol/L	1.7 ± 1.1	1.8 ± 1.2	.32
HDL baseline, mmol/L	1.1 ± 0.4	1.1 ± 0.3	.34
LDL baseline, mmol/L	2.4 ± 0.9	2.3 ± 0.9	.39
Systolic BP baseline, mmHg	128.7 ± 16.4	124.5 ± 17.6	.16
Diastolic BP baseline, mmHg	78.5 ± 10.5	76.0 ± 9.6	.16
Body weight baseline, kg	84.7 ± 10.8	82.9 ± 12.0	.26
BMI baseline, kg/m^2^	30.9 ± 3.3	31.7 ± 3.5	.18
Creatinine, μmol/L	70.2 ± 13.8	72.1 ± 14.4	.60
eGFR, mL/min/L	60.9 ± 0.4	60.6 ± 2.6	.56
CNFD, fibers/mm^2^	24.3 ± 8.6	27.2 ± 9.2	.10
CNBD, branches/mm^2^	59.0 ± 35.3	64.3 ± 39.5	.28
CNFL, mm/mm^2^	16.4 ± 6.4	17.8 ± 6.3	.19
VPT, V	7.7 ± 3.6	10.8 ± 7.2	<.01
ESC feet, μS	58.9 ± 11.2	59.2 ± 20.5	.47
DN4, score	1.4 ± 1.7	3.3 ± 2.6	<.0001
Burning pain, %	9.1	47.2	<.01
Numbness, %	40.9	66.0	<.05

*Note*: Numeric variables are summarized as mean ± standard deviation and compared using independent *t*‐test.

Abbreviations: BP, blood pressure; CNBD, corneal nerve branch density; CNFD, corneal nerve fiber density; CNFL, corneal nerve fiber length.

At follow‐up (Table 3), participants in the WG group showed a significant increase in systolic blood pressure (*p* = .01) and HDL (*p* < .0001) and a significant decrease in LDL (*p* = .01), but no significant change in body weight (*p* = .11), HbA1c (*p* = .18), and total cholesterol levels (*p* = .18). In contrast, participants in the WL group showed a significant reduction in body weight (85.4 ± 11.4 vs. 78.7 ± 9.6 kg, *p* = .01), BMI (31.1 ± 3.0 vs. 29.8 ± 3.6 kg, *p* < .05), and triglycerides (1.65 ± 1.22 vs. 1.19 ± 0.40 mmol/L, *p* < .05), but no change in HbA1c (*p* = .08), total cholesterol (*p* = .44), or blood pressure (*p* = .07, .21).

**TABLE 3:** Comparison of clinical and neuropathy characteristics between baseline and last visit in participants with type 2 diabetes on glucose lowering medications associated with weight loss and gain.

	Weight loss group (*n* = 23)				Weight gain group (*n* = 53)				
	Baseline	Last visit	*p* value	Δ	Baseline	Last visit	*p* value	Δ	*p* value for Δ
HbA1c, mmol/mol	63.9 ± 11.5	58.4 ± 13.0	.08	‐5.6 ± 16.6	66.2 ± 9.3	68.1 ± 14.6	.18	1.9 ± 14.1	.17
Hb1Ac, %	8.0 ± 1.1	7.5 ± 1.2	.08	−0.5 ± 1.5	8.2 ± 0.8	8.4 ± 1.3	.18	0.2 ± 2.0	.17
Total cholesterol, mmol/L	4.3 ± 1.3	4.3 ± 1.3	.44	0.1 ± 1.9	4.2 ± 1.2	4.09 ± 0.9	.18	−0.1 ± 1.1	.58
Triglyceride, mmol/L	1.7 ± 1.2	1.2 ± 0.4↓	<.05	−0.5 ± 1.1	1.8 ± 1.3	1.86 ± 1.1	.42	0.0 ± 1.6	.19
HDL, mmol/L	1.2 ± 0.3	1.3 ± 0.4	.07	0.1 ± 0.4	1.1 ± 0.3	1.26 ± 0.4↑	<.0001	0.2 ± 0.3	.82
LDL, mmol/L	2.3 ± 0.9	2.2 ± 0.8	.25	−0.1 ± 0.9	2.3 ± 0.9	2.04 ± 0.8↓	<.01	−0.3 ± 0.9	.61
Systolic BP, mmHg	127.7 ± 16.1	131.6 ± 14.0	.21	3.9 ± 21.9	124.4 ± 17.9	130.4 ± 14.3↑	<.01	6.0 ± 19.6	.68
Diastolic BP, mmHg	78.0 ± 10.5	74.0 ± 7.7	.07	−4.0 ± 12.3	75.9 ± 9.8	74.9 ± 8.1	.27	−1.0 ± 12.1	.34
Body weight, kg	85.4 ± 11.4	78.7 ± 9.6↓	<.01	−6.7 ± 11.7	82.7 ± 12.4	81.1 ± 11.1	.11	−3.3 ± 14.6	.36
BMI, kg/m^2^	31.1 ± 3.0	29.8 ± 3.6	<.05	−3.0 ± 7.8	32.0 ± 3.3	31.7 ± 3.9	.28	−1.4 ± 6.1	.36
CNFD, fibers/mm^2^	24.3 ± 8.6	24.5 ± 9.2	.44	0.2 ± 7.4	27.2 ± 9.2	22.0 ± 7.8↓	<.0001	−5.2 ± 6.9	<.01
CNBD, branches/mm^2^	59.0 ± 35.3	40.1 ± 28.7↓	<.01	−18.9 ± 28.5	64.3 ± 39.5	36.6 ± 24.0↓	<.0001	−27.7 ± 36.4	.31
CNFL, mm/mm^2^	16.4 ± 6.4	15.9 ± 6.2	.34	−0.4 ± 4.8	17.8 ± 6.3	14.2 ± 5.3↓	<.0001	−3.5 ± 4.7	<.01
VPT, V	7.7 ± 3.6	7.4 ± 4.0	.31	−0.3 ± 2.6	10.1 ± 5.9	11.1 ± 7.1	.15	1.1 ± 6.0	.20
ESC feet, μS	58.9 ± 11.2	61.6 ± 15.8	.17	2.7 ± 13.1	60.0 ± 19.8	59.6 ± 17.8	.43	−1.6 ± 16.7	.28
DN4, score	1.4 ± 1.7	1.2 ± 1.8	.30	−0.2 ± 2.0	3.3 ± 2.6	2.9 ± 2.2	.11	−0.4 ± 2.4	.75

*Note*: Arrows indicate direction of change. Numeric variables are summarized as means ± standard deviation. Variables were compared using paired *t*‐test. The results include only participants who had both baseline and follow‐up measurements, which may differ from those in Table 2 where all participants' baseline measurements are considered. This reflects a smaller subset due to the availability of follow‐up data.

Abbreviations: Δ, difference; CNBD, corneal nerve branch density; CNFD, corneal nerve fiber density; CNFL, corneal nerve fiber length.

### 3.2 Neuropathy measures between WG and WL groups

At baseline (Table 2), both groups had similar corneal nerve morphology (*p* = .10, .28) and sudomotor function (*p* = .47). However, the WG group had a significantly higher vibration perception threshold (VPT) (10.8 ± 7.2 vs. 7.7 ± 3.6 V, *p* = .01) and more neuropathic symptoms (3.3 ± 2.6 vs. 1.4 ± 1.7, *p* < .0001) compared with the WL group.

**FIGURE 1:**
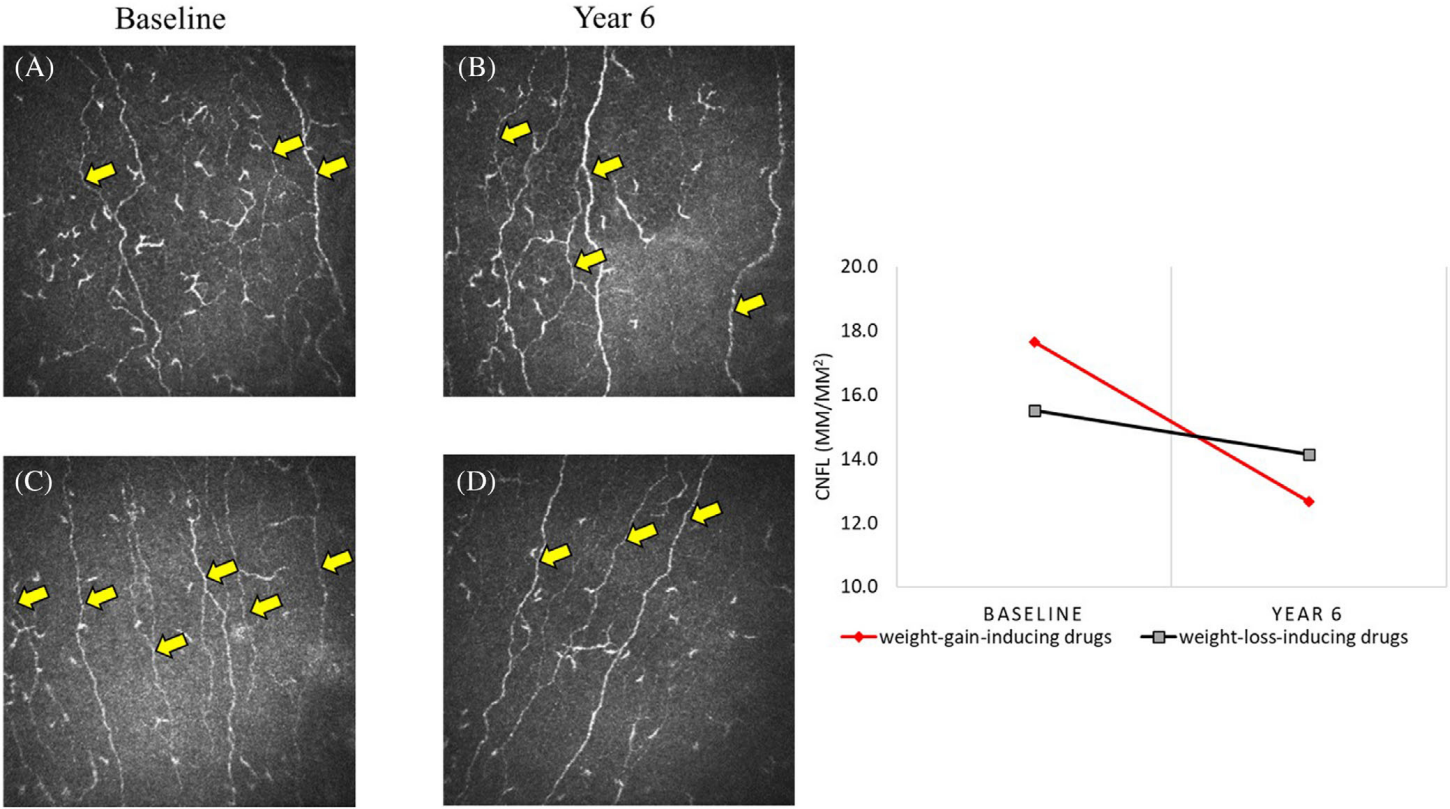
Corneal confocal microscopy images and CNFL changes over 6 years in participants with type 2 diabetes on glucose lowering medications associated with weight loss or weight gain. Images of corneal nerve fibers (yellow arrows) in a participant treated with medications associated with weight loss (WL) at baseline (A) and at 6‐year follow‐up (B) and in a participant treated with medication associated with weight gain (WG) at baseline (C) and at 6‐year follow‐up (D) demonstrating a reduction in nerve fibers at follow‐up. The accompanying line graph compares change in corneal nerve fiber length (CNFL) from baseline to year 6 in participants on WL medications (black line) and WG medications (red line), demonstrating a greater reduction in the WG group.

At follow‐up (Table 3; Figure 1), participants in the WG group showed a significant reduction in corneal nerve fiber density (CNFD) (27.2 ± 9.2 vs. 22.0 ± 7.8 fibers/mm^2^, *p* < .0001), corneal nerve branch density (CNBD) (64.3 ± 39.5 vs. 36.6 ± 24.0 branches/mm^2^, *p* < .0001), and corneal nerve fiber length (CNFL) (17.8 ± 6.3 vs. 14.2 ± 5.3 mm/mm^2^, *p* < .0001). In contrast, those in the WL group showed a reduction in CNBD only (59.0 ± 35.3 vs. 40.1 ± 28.7 branches/mm^2^, *p* < .01) with no change in CNFD or CNFL (*p* = .34–.44). The change in CNFD (0.2 ± 7.4 vs. −5.2 ± 6.9 fibers/mm^2^, *p* < .01) and CNFL (−0.4 ± 4.8 vs. −3.5 ± 4.7 mm/mm^2^, *p* = .01) was significantly different in the WL compared with WG group. Using ANCOVA to adjust for baseline CNFD, CNFL, variation in follow‐up time, and duration of diabetes, the differences in change of CNFD (*F*‐statistic at 5.1, *p* < .05) and CNFL (*F*‐statistic at 4.9, *p* < .05) remained significant between the WG and WL groups, with the WL group showing a change of 0.2 ± 7.4 fibers/mm^2^ for CNFD and −0.4 ± 4.8 mm/mm^2^ for CNFL versus −4.7 ± 6.6 fibers/mm^2^ and −3.6 ± 4.7 mm/mm^2^ in the WG group, respectively. In both groups, there was no change in VPT (*p* = .15–.31), sudomotor function (*p* = .17–.43), and neuropathic symptoms (*p* = .11–.30). The results in Table 3 are based on participants with both baseline and follow‐up data, representing a smaller subset compared with Table 2, where all baseline measurements are included.

### 3.3 Association of CCM measures with weight‐modifying medications (Table 4)

**TABLE 4:** Associations of CCM measures with weight‐modifying medications.

Dependent variable	Independent variable	Coefficient	95% CI	*p* value
ΔCNFD, fibers/mm^2^	Weight gain inducing GL medications	−4.7	−8.2 to −1.2	<.01
	ΔBMI, kg/m^2^	−0.2	−0.4 to 0.01	.06
	Δtriglyceride, mmol/L	−1.2	−2.3 to −0.04	<.05
ΔCNBD, branches/mm^2^	Weight gain inducing GL medications	−8.8	−25.8 to 8.3	.31
ΔCNFL, mm/mm^2^	Weight gain inducing GL medications	−3.0	−5.3 to −0.6	<.01
	ΔBMI, kg/m^2^	−0.1	−0.2 to 0.1	.23

Abbreviations: Δ, difference; CNBD, corneal nerve branch density; CNFD, corneal nerve fiber density; CNFL, corneal nerve fiber length; GL, glucose lowering.

CNFD decreased significantly by 5.5 fibers/mm^2^ (95% CI: −9.0 to −2.0, *p* < .01) in the WG group and reduced CNFD remained significantly associated with the use of medications inducing WG (*β* = −4.7 fibers/mm^2^, 95% CI: −8.2 to −1.2, *p* < .01) after adjusting for changes in BMI and triglycerides. CNFL decreased significantly by 3.1 mm/mm^2^ (95% CI: −5.5 to −0.8, *p* = .01) in the WG group and reduced CNFL remained significantly associated with the use of medications inducing WG after adjusting for change in BMI (*β* = −.3 mm/mm^2^, 95% CI: −5.3 to −0.6, *p* = .01). Change in CNBD showed no association with medications associated with WG (*β* = −8.8 branches/mm^2^, 95% CI: −25.8 to 8.3, *p* = .31).

## 4 DISCUSSION

This study shows that progressive corneal nerve loss is associated with glucose lowering treatment associated with WG. At baseline, those on WG medication reported more neuropathic symptoms and had a higher vibration perception threshold, but comparable corneal nerve morphology to those on WL medications. Over a 56‐month period, participants on WG medications showed no significant changes in body weight and triglyceride levels but a decline in all three corneal nerve measures, with no change in neuropathic symptoms, vibration perception, or sudomotor function, while participants on WL medications had significant weight loss and reduction in triglycerides, with no change in corneal nerve fiber density or length, although with a decline in corneal nerve branch density.

In our previous study,^11^ over 24 months, we found that glucose lowering medication associated with WG was associated with a reduction in CNBD, but with no change in corneal nerve measures in those on glucose lowering medication associated with WL. In this 56‐month extension study of the same T2D cohort, medications associated with WG led to a global reduction in CNFD, CNBD, and CNFL independent of WG. In contrast, in those on glucose lowering medication associated with WL, while there was a reduction in CNBD, there was no change in CNFD or CNFL, indicative of a potential neuroprotective effect. This contrasts with our previous clinical trial which showed that both basal‐bolus insulin and a combination of once weekly exenatide with pioglitazone promoted corneal nerve regeneration independent of changes in HbA1c, body weight, or lipid profiles.^17^ However, in that study, there was a marked improvement in HbA1c of approximately 3% and weight gain in both groups. This difference might be attributed to the trial targeting T2D participants with poor glycemic control, where the more marked improvement in HbA1c may have promoted nerve regeneration.

Obesity increases the risk of developing DPN through increased insulin resistance, impaired glucose metabolism, dyslipidemia, and inflammatory cytokines.^10^, ^25^, ^26^, ^27^, ^28^ Weight loss after bariatric surgery is associated with corneal nerve regeneration and an improvement in other neuropathy measures in individuals with T2D and obesity^12^ and obesity without diabetes.^29^ Our study shows that glucose lowering medication associated with weight loss leads to a reduction in body weight and triglycerides and prevents loss of corneal nerve fibers, which may benefit DPN. The beneficial effects of glucose lowering medication associated with weight loss on DPN are relevant given the shift in the pharmacotherapeutic strategy from a traditional glucocentric to weight‐centric approach. Multiple incretin based therapies, especially semaglutide^30^ and tirzepatide^31^ have demonstrated notable efficacy in the treatment of obesity. Historically, the glucocentric approach has relied on the use of combinations of insulin, thiazolidinediones and sulfonylureas and indeed this is reflected in our study where three out of four participants were prescribed medications that promote WG. The 2023 ADA Standards of Care in Diabetes^13^ now recommends the use of weight‐neutral or WL inducing medications to manage T2D in overweight or obese people, based on their cardiovascular and renal benefit. The findings of our study suggest that such drugs may also confer a benefit for DPN.

In this study, change in HbA1c was not associated with a change in neuropathic symptoms and deficits. Furthermore, despite changes in corneal nerve morphology, there was no change in neuropathic symptoms, vibration perception threshold, or sudomotor function in participants on glucose lowering medication associated with WG or WL. Previously, we found that sustained corneal nerve damage was associated with a progressive increase in both neuropathic symptoms and VPT.^32^ However, we also showed that corneal nerve regeneration occurs within 12 months of simultaneous pancreas and kidney transplantation, while an improvement in neuropathic symptoms and nerve conduction occurred at 24 and 36 months, respectively.^33^ In our previous clinical trial,^17^ both basal‐bolus insulin and a combination of once weekly exenatide and pioglitazone led to significant improvements in HbA1c and corneal nerve regeneration, with no change in vibration perception threshold or sudomotor function. Indeed in a study of twice daily exenatide compared to basal bolus insulin over 18 months, there were no statistically significant difference in the prevalence of clinically confirmed neuropathy, intraepidermal nerve fiber density, measures of cardiac autonomic neuropathy, and nerve conductions studies.^34^ These data highlight the discordance in change of symptoms, signs, and different measures of neuropathy and the inability of most of the currently advocated endpoints to detect an early improvement in DPN.^35^

We acknowledge that the sample size is relatively small, given that only 21 participants were taking medication associated with WL. We also recognize potential socioeconomic differences between participants on WL medications, which are generally more expensive, and those on more affordable WG medications that could indirectly affect health outcomes and treatment adherence, thereby influencing CCM results. Furthermore, those on insulin in the WG medication group are likely to have had T2D for a longer duration, indicating a more advanced stage of diabetes that could exacerbate corneal nerve damage directly or through comorbidities such as obesity, abnormal lipid profiles, and renal impairment. We acknowledge that participants were not randomized to different medications, potentially confounding the outcomes. However, ANCOVA was used to adjust for baseline differences in CNFD, CNFL, follow‐up time, and diabetes duration, and the differences in relation to the change in CNFD and CNFL between groups remained significant. In our analysis, while we assessed the effect of age, diabetes duration, BMI, blood pressure, HbA1c, and lipid profiles, we only adjusted for BMI and triglycerides, as they were significantly associated with CNFD. Participants with renal failure (chronic kidney disease stages 4 and 5) were excluded. Other potential cofounders such as diet, physical activity, and the use of statins or fibrates were not adjusted for. We did not directly assess endothelial function or insulin resistance; both may contribute to neuropathy through impaired blood flow and metabolic dysregulation.^28^, ^36^ The cohort was recruited from a single diabetes clinic, which may limit the representativeness of the sample and increase the potential for selection bias. Future studies with larger, more diverse populations, regular follow‐up intervals, and randomized designs are needed to confirm or refute our findings.

In conclusion, this study suggests that pharmacological interventions for T2D that promote WL may confer benefits for individuals with diabetic neuropathy. Participants taking glucose lowering medications associated with WG exhibited more neuropathic symptoms and deficits at baseline and a greater decline in small nerve fibers, compared with those taking glucose lowering medication associated with WL. Our findings suggest that using medication associated with WL may confer a benefit for DPN in people with T2D.

## AUTHOR CONTRIBUTIONS

Rayaz A. Malik and Georgios Ponirakis were responsible for the study concept and design. All the authors contributed to the acquisition, analysis or interpretation of data. The drafting of the manuscript was done by Rayaz A. Malik and Georgios Ponirakis. For critical revision of the manuscript concerning important intellectual content, every author played a part. The statistical analysis was conducted by Georgios Ponirakis and Ziyad R. Mahfoud. Funding was obtained by Stephen L. Atkin and Rayaz A. Malik. Administrative, technical or material support was provided by all authors. Every author has reviewed and approved the final version of the manuscript, ensuring accountability for all aspects of the work. Rayaz A. Malik, Georgios Ponirakis and Stephen L. Atkin hold access to all study data, ensuring its integrity and the accuracy of the data analysis. Rayaz A. Malik is the guarantor of this work.

## FUNDING INFORMATION

This work was suported by Qatar National Research Fund, Funding ID: BMRP‐5726113101, NPRP 8‐315‐3‐065.

## CONFLICT OF INTEREST STATEMENT

The authors declare no conflicts of interest.

## Data Availability

Coded data from this study are available under data transfer agreement to any researcher. The corresponding author may be contacted to request access.
